# Optimizing HIV treatment through point-of-care CD4 testing – ACHAP Botswana community-based pilot

**DOI:** 10.1186/1471-2334-14-S2-P73

**Published:** 2014-05-23

**Authors:** Kenneth Mugisha, Frank Mwangemi, Jerome Mafeni, Makadzange Panganai, Lesego Busang, Juliana Cuervo Rojas, Ntumba Kanayi, Lesedi Tsalaile, Madisa Mine, Lucy Mpfumi, Mulamuli Moyo

**Affiliations:** 1 African Comprehensive HIV/AIDS Partnerships (ACHAP), Gaborone, Botswana; 2 Ministry of Health, Gaborone, Botswana; 3 Laboratory Technologist Botswana Harvard HIV/AIDS Institute, Botswana

## Introduction

At 17.6%, Botswana has one of the highest HIV prevalence rates globally. Tutume district, has a disproportionately higher HIV prevalence at 20.7% (BAIS, 2008). Only 54% of the infected actually know their HIV status. The investigators set out to determine if increased access to HIV testing and point-of-care CD4 testing can minimize delays to ART initiation.

## Materials and methods

Six ART-providing clinics were allocated 2 PIMA machines each. The PIMA machines were validated against the FACS Calibur machine as the instrument of reference. Community mobilizers were identified for each village. Door-to-door HCT and CD4 testing was done. Community outreaches and HIV/CD4 testing in schools, brick laying factories and mines were organized. Road shows were conducted to attract youth to test. External quality assurance was done quarterly. Data was recorded on HCT registers and Point-of-Care CD4 registers and entered into CSPro database and analyzed using STATA 10.0.

## Results

A total of 6274 clients were tested for HIV in 3 months. Of these, 62 % were new testers, while 501 clients tested HIV positive (8%). Of these, 157 (31.3%) were enrolled on ART. A total of 2717 CD4 tests were performed. Of these, 73.4% were conducted at home, or outreach sites. Majority (65%) of the new testers were women aged 15- 49 years. Youths preferred to test at night during moonlight testing. There were no statistically significant differences in the CD4+ T cell counts obtained using the PIMA with capillary blood and those observed using FACS Calibur and venous blood. The difference tended to be significant at CD4+ T cell counts greater than 350 cell / µl (-21.2 (95% CI -45.3, +2.9)) whereby the PIMA gave slightly lower values than the FACS Calibur. Time from HIV testing to ART-initiation was reduced from 6 to 3 weeks.

## Conclusions

Point-of-care CD 4 testing has the potential to reduce the time to ART initiation; thereby contributing to reduction in morbidity and mortality. Youth-friendly services can contribute to increased HCT uptake in this age group.

